# Evaluation with a haemodynamic simulator undergoing mitral transcatheter edge-to-edge repair in a giant left atrium

**DOI:** 10.1093/ehjcr/ytaf261

**Published:** 2025-05-23

**Authors:** Yusuke Watanabe, Akihisa Kataoka, Kento Kito, Takuya Nishikawa, Keita Saku

**Affiliations:** Department of Cardiology, Teikyo University School of Medicine, 2-11-1 Kaga, Itabashi Ku, Tokyo 173-8606, Japan; Department of Cardiology, Teikyo University School of Medicine, 2-11-1 Kaga, Itabashi Ku, Tokyo 173-8606, Japan; Department of Cardiology, Teikyo University School of Medicine, 2-11-1 Kaga, Itabashi Ku, Tokyo 173-8606, Japan; Department of Research Promotion and Management, National Cerebral and Cardiovascular Center Research Institute, 5-7-1 Fujishiro-dai, Suita-shi, Osaka 565-8565, Japan; Department of Cardiovascular Dynamics, National Cerebral and Cardiovascular Center Research Institute, 5-7-1 Fujishiro-dai, Suita-shi, Osaka 565-8565, Japan

**Keywords:** Transcatheter edge-to-edge repair, Mitral regurgitation, Atrial fibrillation, Giant left atrium, Cardiac simulator

## Case description

A 68-year-old woman with atrial fibrillation and a giant left atrium (LA) underwent transcatheter edge-to-edge repair (TEER) for severe atrial functional mitral regurgitation (MR) (see Supplementary material online, *Video S1–S4*). Although mitral regurgitation improved, she developed hypotension due to iatrogenic mitral stenosis. A cardiovascular simulator visualized impaired left ventricular (LV) filling in this highly compliant LA, supporting the physiological mechanism behind post-procedural instability.

**Figure 1:**
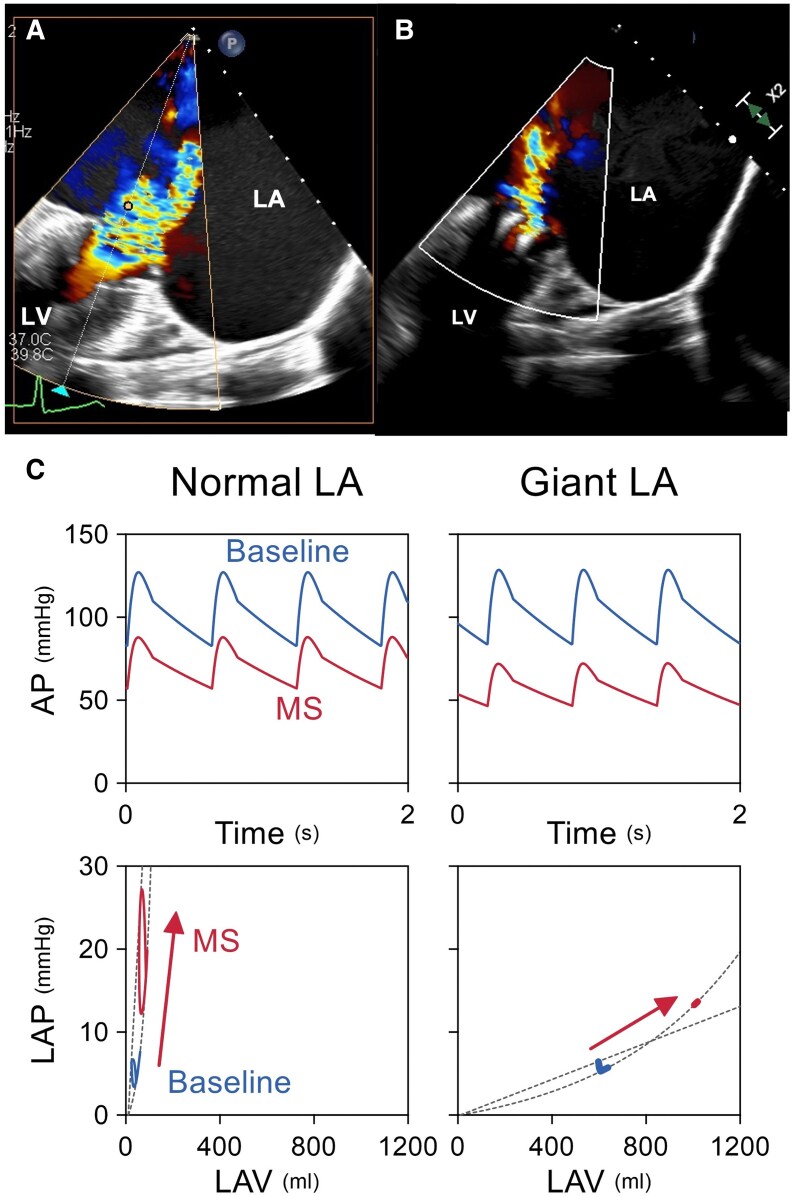
(*A*, *B*) Transesophageal echocardiography images pre (*A*) and post-transcatheter edge-to-edge repair in a giant left atrium (*B*). (*C*) Giant left atrium prevents the increase in LV filling, resulting in a significant decrease in cardiac output. TEE, transesophageal echocardiography. TEE, transesophageal echocardiography; TEER, post-transcatheter edge-to-edge repair; LA, left atrium.

Severe MR with markedly enlarged LA (100 mm) was confirmed on echocardiography. TEER with two MitraClips reduced MR to mild-moderate (*Figure 1A*, Supplementary material online, *Video S5*) but raised mitral gradient to 6 mmHg. Hypotension persisted post-procedure. Cardiovascular simulation confirmed that impaired LA pressure build-up contributed to reduced LV filling and hypotension (*Figure 1B* and *C*). This case highlights the haemodynamic challenges of TEER in a giant LA. Unlike a normal-sized LA, a giant LA’s high compliance prevents effective pressure generation, impairing LV filling under mitral stenosis conditions. Transcatheter edge-to-edge repai in giant LA cases can lead to iatrogenic MS and haemodynamic instability. Cardiovascular simulation provided valuable insights into haemodynamic changes, enhancing understanding of post-TEER effects (see Supplementary material).

## Supplementary Material

ytaf261_Supplementary_Data

## Data Availability

The data underlying this article are available in the article.

## Supplementary material

**Consent:** The author confirms that written consent for submission and publication of this case report, including images and associated text, has been obtained from the patient in line with COPE guidance.

**Funding:** None declared.

