# Supplementary material for: Evaluation with a haemodynamic simulator undergoing mitral transcatheter edge-to-edge repair in a giant left atrium
Source: Eur Heart J Case Rep. 2025 May 23;9(6):ytaf261. doi: 10.1093/ehjcr/ytaf261 (PMC12149846; doi:10.1093/ehjcr/ytaf261)
Supplement: ytaf261_Supplementary_Data [file ytaf261_supplementary_data.zip › Supplementals combined.docx]

**­­****Computer simulation of giant LA**

We have developed a cardiovascular simulator (Fig. S1) using MATLAB/Simulink 2024b (Mathworks, Massachusetts, USA). In the simulator, the four cardiac chambers were represented by a time-varying elastance. The systemic and pulmonary circulations were represented by resistance-compliance circuits. The four cardiac valves were represented as functional resistance.

*Modelling of the heart*

Time-varying elastance [E(t)] in each chamber determines the relationship between instantaneous chamber pressure [P(t)] and instantaneous chamber volume [V(t)].

$$\begin{aligned} P\left( t \right)=P_{\mathrm{ed}}\left( V \right)+E\left( t \right)\left[ P_{\mathrm{es}}\left( V \right)-P_{\mathrm{ed}}\left( V \right) \right] \#\left( C1 \right) \end{aligned}$$

in which:

$$\begin{aligned} P_{\mathrm{ed}}\left( V \right)=\alpha{(e}^{\beta\left( V-V_{0} \right)}-1) \#\left( C2 \right) \end{aligned}$$

$$\begin{aligned} P_{\mathrm{es}}\left( V \right)=E_{\mathrm{es}}\left( V-V_{0} \right) \#\left( C3 \right) \end{aligned}$$

and

$$\begin{aligned} E\left( t \right)=\left\{ \begin{matrix} \frac{1}{2}\left\{ \sin\left[ \left( \frac{\pi}{T_{\max}} \right)t-\frac{\pi}{2} \right]+1 \right\} & 0<t\leq\frac{4}{3}T_{\max} \\ \frac{3}{4}e^{-\frac{\left( t-\frac{4}{3}T_{\max} \right)}{\tau}} & t>\frac{4}{3}T_{\max} \end{matrix} \right. \#\left( C4 \right) \end{aligned}$$

where P_ed_(V) is end-diastolic pressure as a function of volume, P_es_(V) is end-systolic pressure as a function of volume, V_0_ is unstressed volume, E_es_ is end-systolic elastance, α and β are constants of the end-diastolic pressure–volume relationship, t is the time from the start of the systole, T_max_ is the point of maximal chamber elastance, and τ is the time constant of relaxation. All values are described in table S1.

*Modelling of valves*

We modeled cardiac valves by modified from a Bernoulli's principle based on previous report (1).

$$\begin{aligned} Q=50.4\cdot c\cdot A\cdot\sqrt{\Delta P} \#\left( C5 \right) \end{aligned}$$

where c is the flow constant with a value of 0.75, A is the valve area, and △P is the pressure gradient across the valve.

*Modelling of atrial fibrillation*

Atrial fibrillation (AF) was simulated only as loss of atrial contraction (E_es_ = 0.) while maintaining regular rhythm. Irregular rhythmic contractions were not performed because they cause hemodynamics to change with each heartbeat, making evaluation difficult.

*Modelling of vascular system*

Both the systemic and pulmonary vascular systems were modeled using a 7-elemennt resistance–capacitance network model, consisting of lumped arterial, capillary, and venous capacitances (C_A_, C_C_, and C_V_), characteristic impedance (R_Z0_), and lumped arterial, capillary, and venous resistances (R_A_, R_C_, and R_V_) (Fig. S1). All values are described in table S2.

*Parameter settings*

As shown in table S3, diastolic component β, end-systolic elastance and stressed blood volume (SBV) were set to simulate giant LA. Left and right atrium Ees were set to simulate AF. Mitral regurgitation (MR) area was set to simulat MR. Mitral valve area was set to simulate MS.

1. J.D. Thomas, A.E. Weyman, Fluid dynamics model of mitral valve flow: Description with in vitro validation, Journal of the American College of Cardiology 13 (1989) 221–233. https://doi.org/10.1016/0735-1097(89)90575-5.

Fig. S1


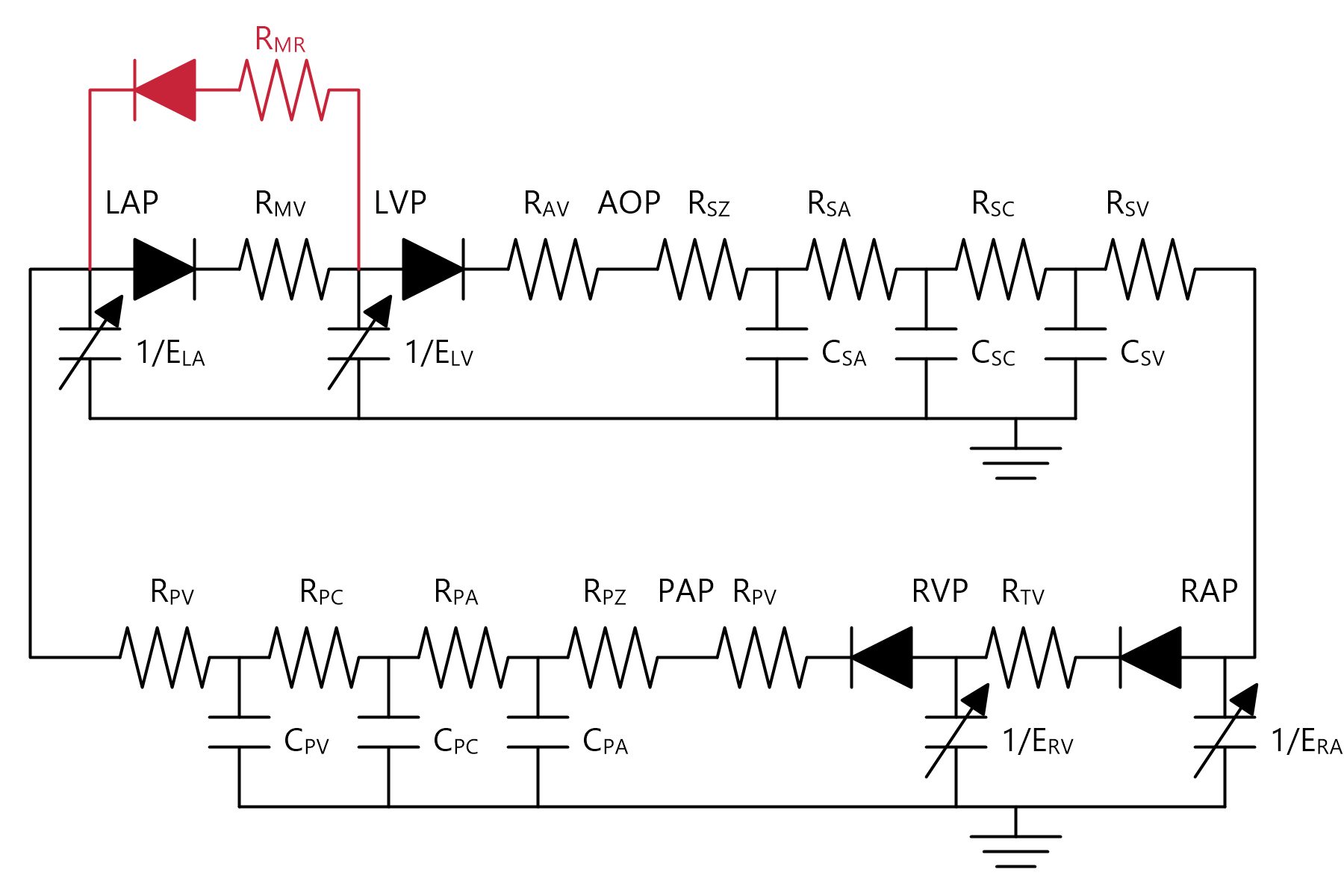


Circuit diagram of cardiovascular simulation model. LAP, left atrial pressure; LVP, left ventricle pressure; AP, arterial pressure; CVP, central venous pressure; E_LA_, time varying elastance of left atrium; D_MV_, mitral valve; R_MV_, resistance of mitral valve; R_MR_, resistance of mitral valve regurgitation; E_LV_, time varying elastance of left ventricle; D_AV_, aortic valve; R_AV_, resistance of aortic valve; R_SZ0_, characteristic impedance of systemic circulation; C_SA_, compliance of systemic circulation; R_SA_, resistance of systemic artery; C_SC_, compliance of systemic capillary vessels; R_SC_, resistance of systemic capillary vessels; C_SV_, compliance of systemic vein; R_SV_, resistance of systemic vein; PAP, pulmonary artery pressure; RVP, right ventricular pressure; RAP, right atrial pressure; R_PV_; resistance of pulmonary vein; C_PV_, compliance of pulmonary vein; R_PC_, resistance of pulmonary capillary vessels; C_PC_, compliance of pulmonary capillary vessels; R_PA_, resistance of pulmonary artery; C_PA_, compliance of pulmonary artery; R_PZ0_, characteristic impedance of pulmonary circulation; R_PAV_, resistance of pulmonary valve; D_PAV_, pulmonary valve; E_RV_, time varying elastance of right ventricle; R_TV_, resistance of tricuspid valve; D_TV_, tricuspid valve; E_RA_, time varying elastance of right atrium.

Table S1. Parameters of heart

|  | LA | LV | RA | RV |
| --- | --- | --- | --- | --- |
| E_es_ (mmHg/ml) | – | 2.1 | – | 0.5 |
| α | 3 | 1.4 | 1.8 | 0.9 |
| β | – | 0.015 | 0.025 | 0.015 |
| V_0_ (ml) | 10 | 10 | 10 | 10 |
| T_max_ (ms) | 150 | 250 | 150 | 250 |
| τ (sm) | 30 | 20 | 30 | 20 |

LA, left atrium; LV, left ventricle; RA, right atrium; RV, right ventricle; E_es_, end-systolic elastance; V_0_, volume axis; T_max_, time to maximum elastance; τ, time constant.

Table S2. Parameters of vascular system

| Compliance (ml/mmHg) | | Resistance (mmHg/ml/s) | | Other parameters | |
| --- | --- | --- | --- | --- | --- |
| C_SA_ | 1.5 | R_SZ0_ | 0.05 | HR (bpm) | 100 |
| C_SC_ | 90 | R_SA_ | 0.9 |  |  |
| C_SV_ | 30 | R_SC_ | 0.023 |  |  |
| C_PA_ | 8 | R_SV_ | 0.023 |  |  |
| C_PC_ | 10 | R_PZ0_ | 0.05 |  |  |
| C_PV_ | 4.5 | R_PA_ | 0.08 |  |  |
|  |  | R_PC_ | 0.023 |  |  |
|  |  | R_PV_ | 0.023 |  |  |

C_SA_, compliance of systemic artery; C_SC_, compliance of systemic capillary vessels; C_SV_, compliance of systemic vein; C_PA_, compliance of pulmonary artery; C_PC_, compliance of pulmonary capillary vessels; C_PV_, compliance of pulmonary vein; R_SZ0_, characteristic impedance of systemic circulation; R_SA_, resistance of systemic artery; R_SC_, resistance of systemic capillary vessels; R_SV_, resistance of systemic vein; R_PZ0_, characteristic impedance of pulmonary circulation; R_PA_, resistance of pulmonary artery; R_PC_, resistance of pulmonary capillary vessels; R_PV_, resistance of pulmonary vein; HR, heart rate.

Table S3. Variable parameters

|  |  | LA  E_es_ (mmHg/ml) | RA  E_es_ (mmHg/ml) | LA  β | MR Area (cm^2^) | MV  Area  (cm^2^) | SBV  (ml) |
| --- | --- | --- | --- | --- | --- | --- | --- |
| Case simulation (Fig. 5) | |  |  |  |  |  |  |
|  | Baseline | 0.5 | 0.2 | 0.025 | 0 | 10 | 1500 |
|  | Giant LA | 0.11 | 0.2 | 0.0017 | 0 | 10 | 2100 |
|  | Giang LA, AF | 0 | 0 | 0.0017 | 0 | 10 | 2100 |
|  | Giant LA, AF, MR | 0 | 0 | 0.0017 | 0.7 | 10 | 2100 |
|  | Giant LA, AF, MR, MS | 0 | 0 | 0.0017 | 0.4 | 2.5 | 2100 |
| MS with/without Giant LA (Visual summary A) | |  |  |  |  |  |  |
|  | Baseline | 0.5 | 0.2 | 0.025 | 0 | 10 | 1500 |
|  | Baseline with MS | 0.5 | 0.2 | 0.025 | 0 | 1 | 1500 |
|  | Giant LA | 0.011 | 0.2 | 0.0017 | 0 | 10 | 2100 |
|  | Giant LA with MS | 0.011 | 0.2 | 0.0017 | 0 | 1 | 2100 |

LA, left atrium; RA, right atrium; E_es_, end-systolic elastance; MR Area, mitral valve backward area; MV Area, mitral valve forward area; SBV, stressed blood volume; AF, atrial fibrillation; MR, mitral regurgitation; MS, mitral stenosis.
